# Hunted Woolly Monkeys (*Lagothrix poeppigii*) Show Threat-Sensitive Responses to Human Presence

**DOI:** 10.1371/journal.pone.0062000

**Published:** 2013-04-16

**Authors:** Sarah Papworth, E. J. Milner-Gulland, Katie Slocombe

**Affiliations:** 1 Division of Ecology and Evolution, Imperial College, Silwood Park Campus, Berkshire, United Kingdom; 2 Centre for Environmental Policy, Imperial College, South Kensington, United Kingdom; 3 Department of Psychology, University of York, York, United Kingdom; Université de Strasbourg, France

## Abstract

Responding only to individuals of a predator species which display threatening behaviour allows prey species to minimise energy expenditure and other costs of predator avoidance, such as disruption of feeding. The threat sensitivity hypothesis predicts such behaviour in prey species. If hunted animals are unable to distinguish dangerous humans from non-dangerous humans, human hunting is likely to have a greater effect on prey populations as all human encounters should lead to predator avoidance, increasing stress and creating opportunity costs for exploited populations. We test the threat sensitivity hypothesis in wild Poeppigi's woolly monkeys (*Lagothrix poeppigii*) in Yasuní National Park, Ecuador, by presenting human models engaging in one of three behaviours “hunting”, “gathering” or “researching”. These experiments were conducted at two sites with differing hunting pressures. Visibility, movement and vocalisations were recorded and results from two sites showed that groups changed their behaviours after being exposed to humans, and did so in different ways depending on the behaviour of the human model. Results at the site with higher hunting pressure were consistent with predictions based on the threat sensitivity hypothesis. Although results at the site with lower hunting pressure were not consistent with the results at the site with higher hunting pressure, groups at this site also showed differential responses to different human behaviours. These results provide evidence of threat-sensitive predator avoidance in hunted primates, which may allow them to conserve both time and energy when encountering humans which pose no threat.

## Introduction

Anti-predator responses can incur opportunity costs, reducing time available for feeding and other activities, or physical costs from energy expenditure or injury [1]. These costs can be reduced if prey are able to distinguish between dangerous and non-dangerous individuals of a single predator species. Not all encounters of prey with predators will be predation events. This may occur for a number of reasons; for example because the predator has recently fed, or if the prey gives alarm calls to a stalking predator and disrupts the hunt [2], [3]. If prey respond with anti-predator strategies to all encounters with a particular species, they may incur significant costs, particularly if the predator is common but attacks are infrequent. Prey which can distinguish between dangerous and non-dangerous individuals of a predator species and respond appropriately will reduce the costs of anti-predator behaviour [4]. This threat sensitive predator response, first suggested by Helfman [5], involves the prey altering their response depending on the magnitude of the threat. Threat sensitive predator responses been previously observed in damselfish [5], elephants [6] and larval treefrogs [7]. Helfman's study showed that damselfish showed stronger responses to predatory trumpetfish which were orientated vertically, indicating they were poised to strike, than to the same model orientated horizontally [5]. In general, threat sensitive predator reactions translates to increased responses when the threat, and therefore risk, is greatest.

Humans are an example of a common predator which does not always attack, and so are a predator to which threat sensitive responses would be particularly appropriate. Bates et al. demonstrated a threat sensitive response to humans in hunted elephants [6]. They conducted experiments where elephants were presented with cloths of different colours and scents. When presented with cloth worn by a Masaai (who hunt elephants), the elephants moved further, moved faster and took longer to relax than when they were presented with one worn by an individual from an ethnic group which does not hunt elephants. When encountering unscented cloths, they displayed more aggressively to the red cloths (the traditional colour of Masaai cloaks) than white cloths. We focus on primates because they are often preferred human prey species due to their relatively large size and conspicuousness, and are often vulnerable to overhunting due to their social nature and low rates of population increase [8]. Primates are particularly interesting as they are good candidates for showing a threat-sensitive response to humans, but this has not yet been tested. Humans are also the main predator of some primate species, yet have received relatively little attention as primate predators when compared with carnivorous mammals and raptors [9].

Zuberbühler et al. [10] and Bshary [11] used human model experiments and showed that Diana monkeys (*Cercopithecus diana*) responded to humans cryptically. However, a later study showed Diana monkeys giving vocalisations in response to human models [12]. Arnold et al. [13] also suggest inconsistent reactions to human presence in putty-nose monkeys (*Cercopithecus nititans*) in Nigeria. Response to the presence of a moving human was cryptic behaviour in 16 of 22 experiments, but groups vocalised during the other six experiments. Cryptic trials were excluded from analysis in the paper, with the authors arguing that it was not possible to conclude the monkeys had seen the stimulus if they did not vocalise. However, the same silent response was observed in far fewer cases to other stimuli (moving leopard 0/11, stationary eagle 2/10, stationary leopard 4/21); arguably suggesting the monkeys had detected the human stimulus, but were responding to it cryptically. A study by Croes et al. [14] in Gabon did not find differences between hunting and non-hunting areas in the number of monkey groups which vocalised in response to human presence, but did find that monkeys in areas which experienced hunting pressure were more likely to flee. As human hunters are generally pursuit rather than ambush hunters and may try and hunt any (but not all) desirable prey they encounter [15], it is perhaps surprising that primates should ever give vocalisations or otherwise draw attention to themselves on encountering a human.

There are two possible mechanisms of adaptation to predation, natural selection over evolutionary time or learning within the lifetime of the individual. Zuberbühler and Jenny [16] argue that in comparison with other predators, high levels of human offtake are evolutionarily recent, so primates have no evolved response and hence respond inconsistently. Although this is possible, fear responses have been observed to develop rapidly in response to novel predators that are introduced experimentally and naturally in the wild [17], [18]. Individuals' learning about predation events through personal experience alone cannot explain this rapid acquisition, but social learning about predators has been demonstrated in various species, including fish, birds, marsupials and primates [19]. Given that social learning has been demonstrated in other primate species in a number of contexts [20]–[22], it seems possible that primates may develop and learn adaptive responses to evolutionarily recent predation events, such as humans.

Thus the seemingly inconsistent primate responses to humans could be socially learned threat-sensitive predator responses. Monkeys in these studies could have been responding to additional behavioural cues from the humans which suggested different levels of threat. Cryptic behaviour is likely to be the best anti-predator strategy against human hunters [15], but appropriate responses to other humans may depend on the characteristics of the humans present. For example, if humans are fishing or conducting other activities below the monkeys for some hours, it may be more appropriate to flee immediately and not waste time freezing. Distinguishing between different human behaviours in this way would reduce the costs of primate anti-predator responses to this common predator, but assumes that prey are able to distinguish specific behaviours in a single species, and react appropriately. Although wild prairie dogs [23] and elephants [6] have been shown to distinguish between different types of human, no previous research has been conducted to determine if primates can make this distinction.

### Predictions of the threat sensitivity hypothesis

Here we examine whether primates can use predator behavioural cues to distinguish dangerous and non-dangerous individuals of the same species. The specific study site in the Ecuadorian Amazon was chosen as one that had areas with both high and low hunting pressure, but in which hunting pressure was not so great that primates were extirpated in the hunting area. In particular, we assessed responses to dangerous and non-dangerous humans. Assuming that hunted monkeys respond to human presence in a manner consistent with the threat sensitivity hypothesis, three predictions were tested:

1. Behaviour changes after exposure to human presence, in a manner consistent with a threat response (e.g. in a way that reduces detectability or by fleeing);
2. The type of response is a function of the perceived magnitude of the immediate threat, based on the simulated behavioural characteristics of the human present (hunter, gatherer or researcher);
3. The type of response is a function of the perceived magnitude of the underlying threat; based on differences in prior exposure to different threat types (high and low pressure hunting areas).

In the study system, monkeys are likely to encounter three types of human: hunters, gatherers and researchers, so each trial will simulate the presence of one of these three types of human behaviour. Of these, hunters pose the greatest threat as they are actively searching for prey, and carry lethal weapons. Gatherers do not pose a lethal threat, but may collect resources as part of a mixed group of hunters and gatherers, or return to the community and report the location of the group to hunters [24]. Researchers pose no lethal threat to monkeys, but may follow groups or even on occasion dart monkeys. For this study we make the assumption that woolly monkey encounters with hunters are likely to be lethal, encounters with gatherers may be associated with (time-delayed) lethalness, and encounters with researchers are unlikely to be lethal. On this basis, the threat sensitivity hypothesis therefore predicts the strongest response to hunters, weaker responses to gatherers and the weakest response to researchers. Nevertheless, the expected mortality from an encounter with each of these types of human may not correlate perfectly with threat levels perceived by the monkeys, and in particular, the perceived threat of researchers may increase if an individual or group has experience of biopsy darts. Response to each of these types of human may also vary with the level of hunting pressure or exposure to gatherers and researchers in an area. For example, in areas with lower hunting pressure, primate populations have lower exposure to hunters so they may react inappropriately. Alternately, less cautious primates in areas with higher hunting pressure may be more likely to be hunted, leaving only more cautious individuals in the population.

## Methods

### Ethics statement

Research plans and protocols were reviewed and approved by the Imperial College Research Ethics Committee (approval reference ICREC_9_2_7) and adhered to the United Kingdom's Animals (Scientific Procedures) Act 1986. Research permit 009-DFO-DPO-M was granted by the Ministerio del Ambiente, Provincial de Orellana, Ecuador to work within Yasuní National Park. Experiments were performed with wild monkey groups, and represented interactions which would normally be experienced by groups in the area. Experiments were conducted between November 2009 and August 2010, and only three experiments were conducted in each area.

### Site and species

Experiments on unhabituated monkeys were conducted in Yasuní National Park, Amazonian Ecuador. Two sites 26 km apart were used, one with higher hunting pressure (HP, Yasuní Research Station) and another with lower hunting pressure (LP, Tiputini Biodiversity Station). Hunting has not been observed at the LP site by staff of Tiputini Biodiversity Station, although hunters did report hunting in the surrounding areas (S. Papworth, unpublished data). It is impossible to state that animals at Tiputini Biodiversity Station have not experienced hunting pressure, thus the site is classified as having “low hunting pressure” rather than being “unhunted”. Both sites are used by researchers, though Tiputini Biodiversity Station generally has more researchers present than Yasuní Research Station. The local people in the experimental area are the Waorani. The majority of activities by individuals in the communities are part of a subsistence economy based on small scale farming, hunting and gathering. Traditionally, hunting technology was limited to hardwood spears and blowpipes whose arrows were tipped with curarae poison. These hardwood spears were used to hunt white-lipped peccaries (*Tayassu pecari*), and the blowpipe was used to hunt monkeys and birds. Although children start learning to hunt small birds with half or three-quarter size blowpipes and Waorani hunting is still predominately for subsistence [25], many have changed their hunting methods from traditional spears and blowpipes to guns and dogs [25]–[27]. Hunters are also now hunting species that were previously considered taboo, such as the tapir (*Tapirus terrestris*) [28]. Although men are the main hunters, some women also hunt, though this is usually opportunistically, such as killing animals with a machete when encountered near the community. Many women also accompany their husbands while they hunt. All males over 18 go hunting, although the frequency with which they do this depends on various factors, such as the number of other adults males in their household and their position within the household [24].

Poeppigi's woolly monkeys (*Lagothrix poeppigii*) were used for the experiments as are they are a preferred prey species in the study area [24] and in the Amazon in general [29]–[31]. They experience higher hunting pressure at the HP site, with an estimated hunting offtake of over 200 individuals per year from an area approximately 800km^2^ (derived from Franzen [25]). The average weight of hunted woolly monkeys is 6.1kg [25], and even though harpy eagles and jaguars prey on similar-sized howler monkeys and are likely to prey on woolly monkeys, there is only one published record of non-human predation on woolly monkeys [32]. Woolly monkeys live in large social groups with overlapping territories which spread over large areas during the day [33]. In the study area at the HP site, sub-groups (groups separated by at least 50–100 m and encountered 10 minutes apart, as defined by Derby [34]) average 9.5 individuals, with a population density of 20.4 individuals per km^2^. Sub-groups at the LP site average 7.9 individuals and have a population density of 31.8 individuals per km^2^ [34], although true densities at this site may be far higher (A. Di Fiore, personal communication). Although all individuals in this study are likely partially habituated to the presence of researchers due to the nearby research stations, care was taken that experiments were conducted outside the area in which woolly monkeys have been intensively habituated by Proyecto Primates (https://webspace.utexas.edu/ad26693/www/yasuni/index.html).

### Experimental conditions

A human behaving according to each of three conditions was presented to seven groups over the course of a year, three groups at the HP site and four at the LP site, giving a total of 21 experiments. It was not possible to conduct these experiments on a greater number of groups due to difficulties locating additional groups in other areas at the HP site and the habituation of groups in all other areas at the LP site. At the LP site, we did not conduct a trial on a group if any individual was observed to have a radio collar, indicating they were part of this project.

**Figure 1:**
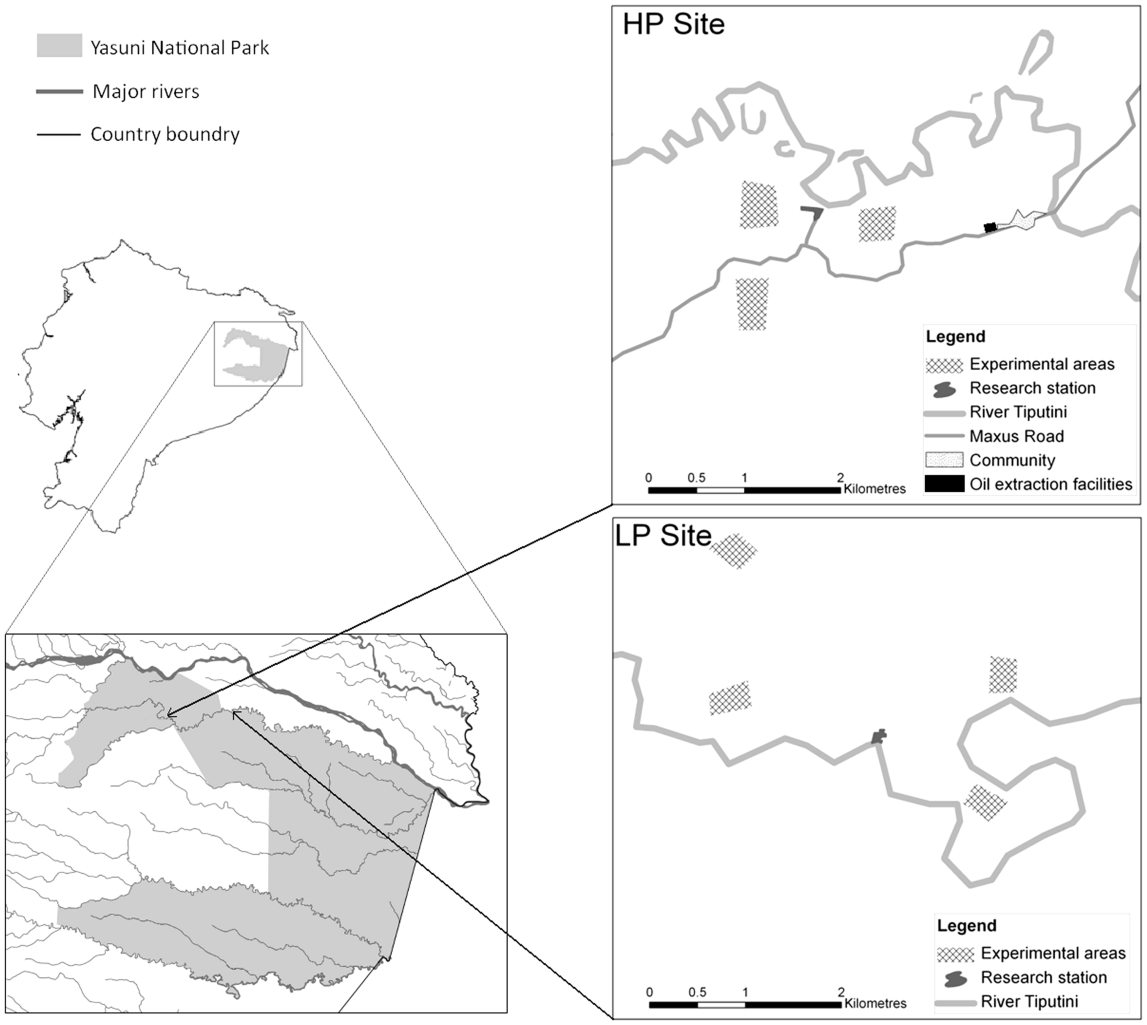
Locations of experiments at the LP and HP sites. One experiment of each type (hunter, gatherer and researcher) was conducted in each of the experimental areas used at the two sites (HP site  =  high hunting pressure, Yasuní Research Centre; LP site  =  low hunting pressure, Tiptutini Biodiversity Station).

To ensure each condition was presented to seven independent groups, one experiment of each type of human behaviour was conducted in each of seven areas. Experiments in the same area were separated by a minimum of nine days (inter-trial duration median  =  69.5 days, range  =  9–199 days). Each area was separated from others by a minimum of 1 km and separation distances less than 1.5 km only occurred when physical barriers such as roads or rivers also existed between locations (Figure 1). It is not inconceivable that woolly monkeys could move across these physical barriers. However, both roads and rivers caused a canopy gap of at least 40 m and often far wider, thus it was assumed social groups did not move across these barriers. During 18 months of fieldwork, woolly monkeys were never observed to cross roads or rivers. During experiments it was not possible to identify individuals, as group members were infrequently visible. Although it is possible that some individuals in each area experienced all three conditions, experiments in a single area were never conducted on the same number of individuals, and experiments recorded group, rather than individual level behaviours.

After the pilot study, it was decided that the use of local assistants was inappropriate – unlike other sites where primates are studied, all local people were involved in hunting, and any local field assistant was likely to return to the location of encountered groups to hunt, which had clear ethical implications for monkeys in the experimental areas and could have a significant impact on the results of later trials. Thus non-local Ecuadorian assistants were used, which, due to the inaccessibility of the sites, reduced the number of possible field assistants to two. This unavoidably led to a high level of pseudo-replication. The low number of field assistants did have the advantage that other characteristics such as sex, age and ethnicity were constant, thus different responses in the experiments can be attributed to behavioural, rather than physical characteristics of the stimulus. The field assistants were aware that experiments were intended to investigate woolly monkey responses to humans. They were not informed of hypothesised differences in response to different humans. Both were aware of the importance of recording accurate observations and knew that both a response and a lack of a response were considered interesting results. The field assistants were not responsible for data recording except contributing to the estimates of height and spread of monkeys during the experiment.

### Experimental procedure

Data from pilot experiments indicated groups took a minimum of 20 minutes after encountering a human to return to baseline behaviour as recorded before human presentation, a 30 minute observation period after the appearance of a human was used, balanced with 30 minutes of baseline data before experimental presentation. On each morning, a target area and condition type was assigned before entering the forest. Groups were located by both sight and sound. On occasion, movement was heard in the trees so SP and the field assistant hid silently nearby in thick vegetation until the species could be confirmed from their vocalisations or by sight. If SP or the field assistant were seen by the monkeys before the experiment started, or made any loud noises, both moved to a location within hearing distance of the group and hid out of sight for two hours before starting the experiment (n = 1). At the start of the experiment, SP and the field assistant hid in dense undergrowth and groups were observed for 30 minutes at a distance of 5–20 metres to determine baseline behaviours before the stimulus was presented. Experiments were abandoned if groups noticed the experimenters in this time. For the experimental presentation, the field assistant walked under the group, behaving as one of the three types of human outlined in Table 1. Key differences between the conditions were silent movement and the presence of a blowpipe for the hunter condition, louder movement and gaze direction away from the monkeys whilst collecting plant material in the gatherer condition, and research equipment, louder movement and gaze direction towards the monkeys for the researcher condition. During the study, the area and experiment type were balanced between two assistants. Therefore, individuals in each of the seven areas could have experienced a maximum of two exposures to a single assistant, and it is unlikely that they would recognise the assistant and that this could affect the outcome of the trial. After five minutes, the field assistant moved away silently and out of sight. It is not possible to know exactly when the monkeys first saw or heard the stimulus (the assistant), but it is assumed that one or more individuals noticed the stimulus within these five minutes. Behaviour was then observed for a further 25 minutes after the removal of the stimulus. Behaviour was recorded using presence/absence of travelling or visibility of any individual in five minute intervals. Height range, spread and number of individuals detected, visibility of individuals and if any individuals had vocalised or travelled (movements greater than 5 m or between trees) were recorded. It was vital that the visibility of the monkeys was assessed from the same spatial location pre and post experimental model presentation. Therefore although the visibility of the monkeys may have been different from alternative viewing angles in the forest, we assessed visibility from the same location throughout the experiment. As experimental conditions and locations were randomly allocated, the probability of failure to see some individuals due to the viewing angle is assumed equal in all experiments and a source of random noise in the observations. Visibility was calculated using the methods of Koné [35], although initial analyses showed that monkeys were only visible in 65 of 252 five minute segments, so a binomial distribution was used as a response, with the group either coded as “visible” or “not visible”. The group was coded as visible if any part of any monkey was visible at any point during a 5 minute segment. All vocal behaviour of the group was recorded with a Marantz PMD661 Professional Portable SD Field Recorder and Seinnheiser ME67 directional microphone. Direction of movement was recorded with a compass and experiment duration with a Casio wristwatch.

**Table 1:** Human behaviour associated with each experimental condition.

	Hunter	Gatherer	Researcher
Equipment	2.4 m blowpipe/50 cm dart quiver	None-collecting leaves/seeds from the forest floor and low shrubs while moving	Small notebook/small bag/binoculars/video camera
Noise level	Very quiet/silent	Normal	Normal
Movement	Slow, aiming the blowpipe at them when directly underneath	Moving from plant to plant below the monkeys, stopping to collect.	Moving around below the monkeys, stopping underneath when directly visible.
Gaze direction	Looking up at monkeys	Looking down and ignoring monkeys	Looking up at monkeys

### Calculations

To calculate the number of vocalisations produced, sound recordings of the experiment were first digitalised, and then cut into five minute segments. In order to allow the five-minute segments to be coded impartially with the coder blind to the condition and period, each five minute segment was initially dummy labelled by the field assistant before the data were coded by SP. Number of vocalisations was determined for each five minute section audibly and confirmed with inspection of the waveform and spectrogram of the sound in the program PRAAT [36]. A more fine-grained analysis of the immediate vocal responses was also conducted, and the number of vocalisations for each minute was calculated for the 5 minute sections immediately before, during and immediately after experimental presentation. As woolly monkey vocalisations are graded and no rigorous description has been produced for wild populations of woolly monkeys, vocalisations were not separated into vocalisation types.

### Baseline differences between sites

During each experiment, it took a median of 30 minutes (range 0–60) to detect individuals in the immediate area and thus estimate the number of individuals likely to detect the stimuli. It is possible that some individuals were not detected during the experiment. Only independently-locomoting animals were included in this estimate. Spread and median height of detected animals were visually estimated during the experiment by SP and the field assistant. Before experiments started, accuracy of visual estimations of distances within the forest were established by comparing visually estimations by both SP and the field assistant with actual distance measured with a 50 m tape. Training continued until distances could be accurately and consistently estimated by both SP and the field assistant. When additional individuals were detected at the periphery of the previously detected sub-group during the experiment, estimated spread among the detected animals increased. Median estimated height also changed when additional individuals were detected. For this reason, height and spread were not included as behavioural measures which could change before and after experimental presentation. The latency in detection of individuals does not, however, change the validity of observations of vocalisations, travelling and visibility. Even if individuals are not immediately detected, they would be detected if they vocalised, travelled or moved to a location where they were visible.

## Analysis

All analyses were conducted using the statistical program R [37]. The number of individuals detected and median estimated height and spread of these individuals before experimental presentation were compared between the two sites using a Wilcoxon rank sum test, which is identical to the Mann-Whitney U test (non-parametric test for comparison of independent samples).

Previous studies on primate responses to humans have used non-parametric analysis methods, and only recorded observed behaviour after the presentation of humans (with the exception of Bshary [11]). This is due in part to these studies comparing loud-calling responses between predator types [10], [13] or because they were observational [14]. This study aims to compare primate antipredator responses to different types of human behaviour, thus the crucial contrast is the change in behaviour from before experimental presentation to afterwards.

As autocorrelation in a single experiment was considered a greater source of potential error than the possibility that some individuals experienced more than one experiment, generalised estimating equations were used for analyses. Generalised estimating equations are semi-parametric regression techniques which perform consistently even under mild violations of the specified variance structure [38], such as data which do not perfectly conform to a Poisson distribution. Subsequent observations in each experiment (five minute sample periods) were likely to be related as members of the group responded to the activity of others (e.g. responding to vocalisations), so generalised estimating equations with an auto-regressive AR1 correlation structure were used. Failing to account for this autocorrelation would increase the chances of a false positive result. For each behaviour measured, the correlation between sequential periods is shown. Correlation is shown as a probability (including the standard error) that an observed behaviour is the same as the previous period. To compare responses to the presence of humans, generalised estimating equations with the package geepack were used [39].Three explanatory variables and their interactions were used for all models and a summary of the implications of including each of these variables and interactions in the final model are given in Table 2.

**Table 2:** Explanatory variables and interactions included in the maximal model and the interpretation of these if included in the final model.

Variable	Interpretation of inclusion in final model
Condition1	Behaviour differs depending on the type of human presented
Period2	Behaviour differs before and after experimental presentation (EP)
Site3	Behaviour differs between sites
Condition x Period	Behaviour before and after EP differs depending on condition
Site x Period	Behaviour before and after EP differs depending on site
Condition x Site x Period	Behaviour before and after EP differs with condition, and these differences also differ between sites.

1 hunter, gatherer or researcher.

2 before and after experimental presentation. Periods are before, during and after for immediate vocal response – see text for more details.

3 HP or LP.

From all possible models nested in the global model, nine models were selected which tested the specific hypotheses of the study. In particular, condition was only included in models in interaction with experimental periosd, as we were interested in changes in behaviour as a result of experimental manipulation and how that varied across stimulus types. Models were compared using QICu, a quasi-likelihood version of AIC which is appropriate to the quasi-likelihood methods of generalised estimating equations. QICu and ΔQICu for all nine models are presented in the supporting information (Table S1, Table S2, Table S3, Table S4). Post-hoc Wald tests were conducted on the best model using the R package contrast [40] to determine which experiment types showed significant behavioural differences between the period before and after experimental presentation. For immediate vocal response, three periods were used: before, during and after experimental presentation. As generalised estimating equations were used, the period during experimental presentation was included to allow continuity for the AR1 correlation structure. Post-hoc Wald tests compared the immediate vocal response in the five minutes before and after experimental presentation as it was not possible to know at which point during the experimental presentation the field assistant was first observed. Difference in behaviour after experimental presentation is graphed on a logit scale of probability for binomial variables and a log scale for Poisson variables in order to display standard errors.

## Results

### Baseline differences in height, group size and spread

Across all experiments, a median of 10 animals were detected during the 30 minutes before experimental presentation (interquartile range 7–15), and this did not differ between sites (Wilcoxon rank sum, N_HP site_ = 9, N_LP site_ = 12, W = 31, P = 0.11). Median height of these detected animals before experimental presentation was 16.50 m (interquartile range 14.17–20.00 m), and no difference between sites was found (Wilcoxon rank sum, N_HP site_ = 9, N_LP site_ = 12, W = 36, P = 0.21). Median estimated spread of individuals was greater at the HP site than the LP site (Wilcoxon rank sum, N_HP site_ = 9, N_LP site_ = 12, W = 89, P = 0.013, median_LP site_ = 55 m, range = 45–70, median_HP site_ = 45 m, range = 35–60).

### Immediate vocal response in five minutes after experimental presentation

**Figure 2:**
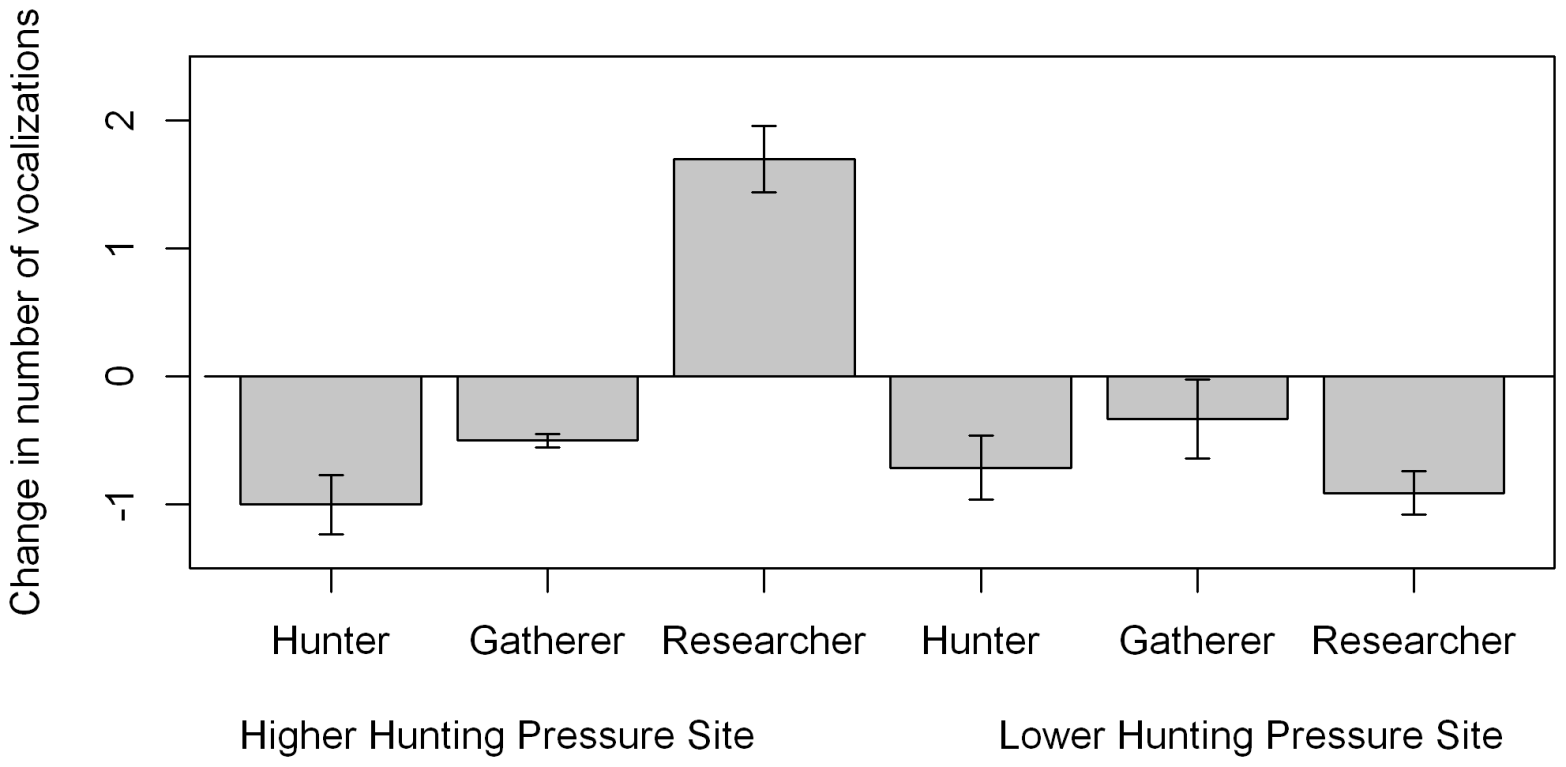
Immediate vocal response in the five minutes after experimental presentation. Change in number of vocalisations (log scale to allow standard errors to be displayed) given in the five minutes immediately after experimental presentation when compared with the five minutes immediately before. Error bars show standard errors of the estimate. P values of differences between the period before and after experimental presentation are shown: * p = 0.05–0.01, **p = 0.01–0.001, ***p<0.001. Hunter condition: Wald test, HP site: Z = 2.42, df = 1, p = 0.016; LP site: Z = 15.3, df = 1, p<0.001. Gatherer condition: HP site: Wald test, Z = 2.8, df = 1, p = 0.005, LP site: Wald test, Z = 6.6, df = 1, p<0.001. Researcher condition: Wald test, p>0.05 for both sites.

To describe immediate vocal response of woolly monkeys to human presentation, the best model included all interactions (Table S1), and significant autocorrelation (0.65±0.11) between sequential observations in the same experiment. After being presented with humans behaving as hunters, vocalisations decreased at both sites, but no significant response to researchers was observed at either site. After presentation of the gatherer condition, vocalisations increased at the LP site but decreased at the HP site (Figure 2).

### Change in number of vocalisations in 30 minutes after experimental presentation

**Figure 3:**
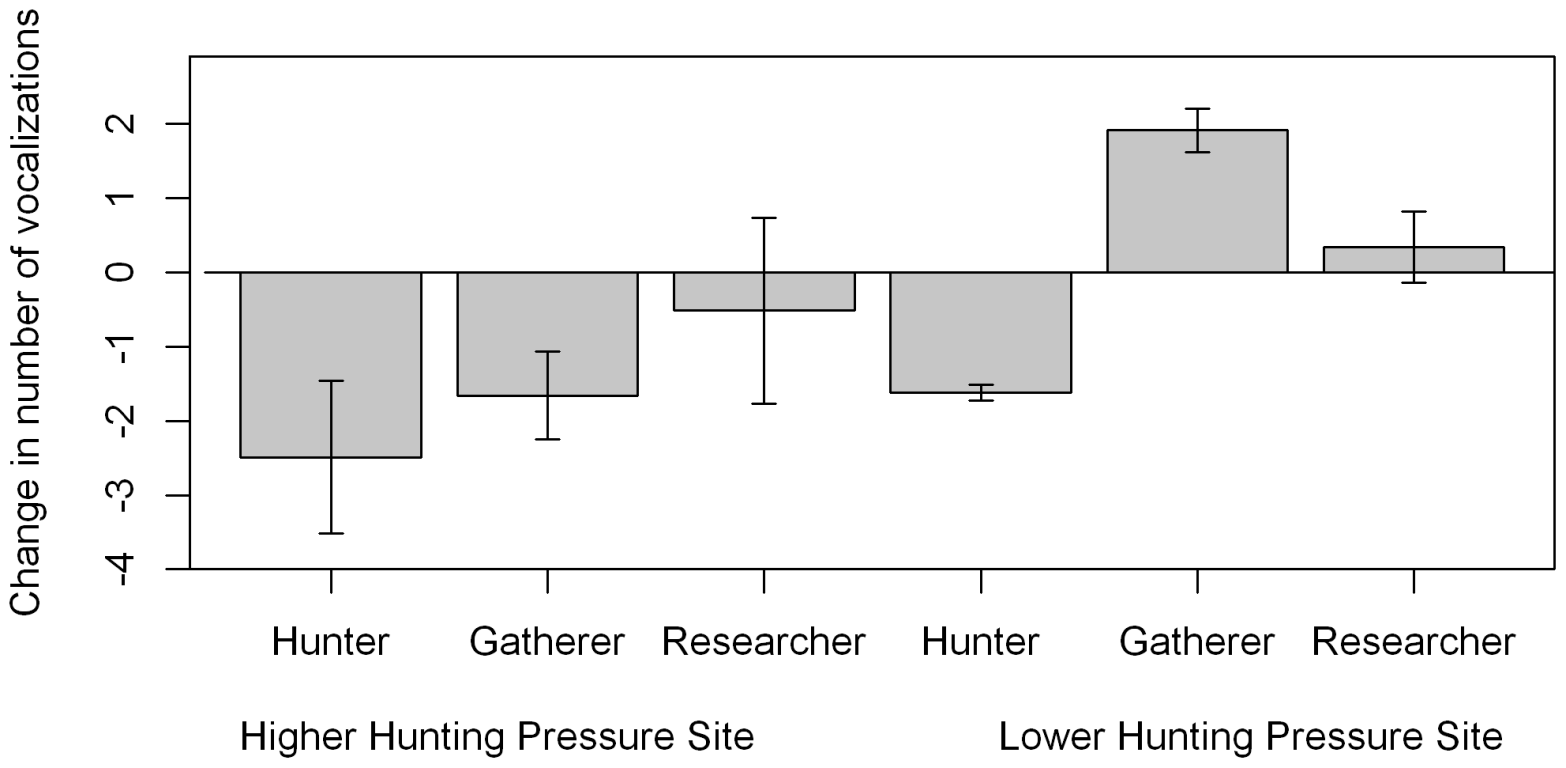
Change in vocal behaviour in the 30 minutes after experimental presentation. Change in number of vocalisations (log scale to allow standard errors to be displayed) given in the 30 minutes after start of experimental presentation, compared with the previous 30 minutes. Error bars show standard errors of the estimate. P values of differences between the period before and after experimental presentation are shown: * p = 0.05–0.01, **p = 0.01–0.001, ***p<0.001. Hunter condition: Wald test, HP site: Z = 4.26, df = 1, p<0.001; LP site: Z = 2.84, df = 1, p = 0.045. Researcher condition: Wald test, HP site: Wald test, Z = 6.53, df = 1, p<0.001, LP site: Z = 5.23, df = 1, p<0.001. Gatherer condition: Wald test, HP site: Z = 10.3, df = 1, p<0.001, LP site, p>0.05.

To describe the number of vocalisations in each five minute block throughout the experiment, the best model included all interactions (Table S2), and a correlation of 0.32±0.13 between sequential observations in the same experiment. After experimental presentation, the number of vocalisations decreased in response to most conditions. Number of vocalisations decreased at both sites after presentation of hunters. After presentation of the researcher condition, number of vocalisations decreased at the LP site but increased at the HP site. In response to the gatherer condition, no response was observed at the LP site, but number of vocalisations decreased at the HP site (Figure 3).

### Change in travelling in 30 minutes after experimental presentation

**Figure 4:**
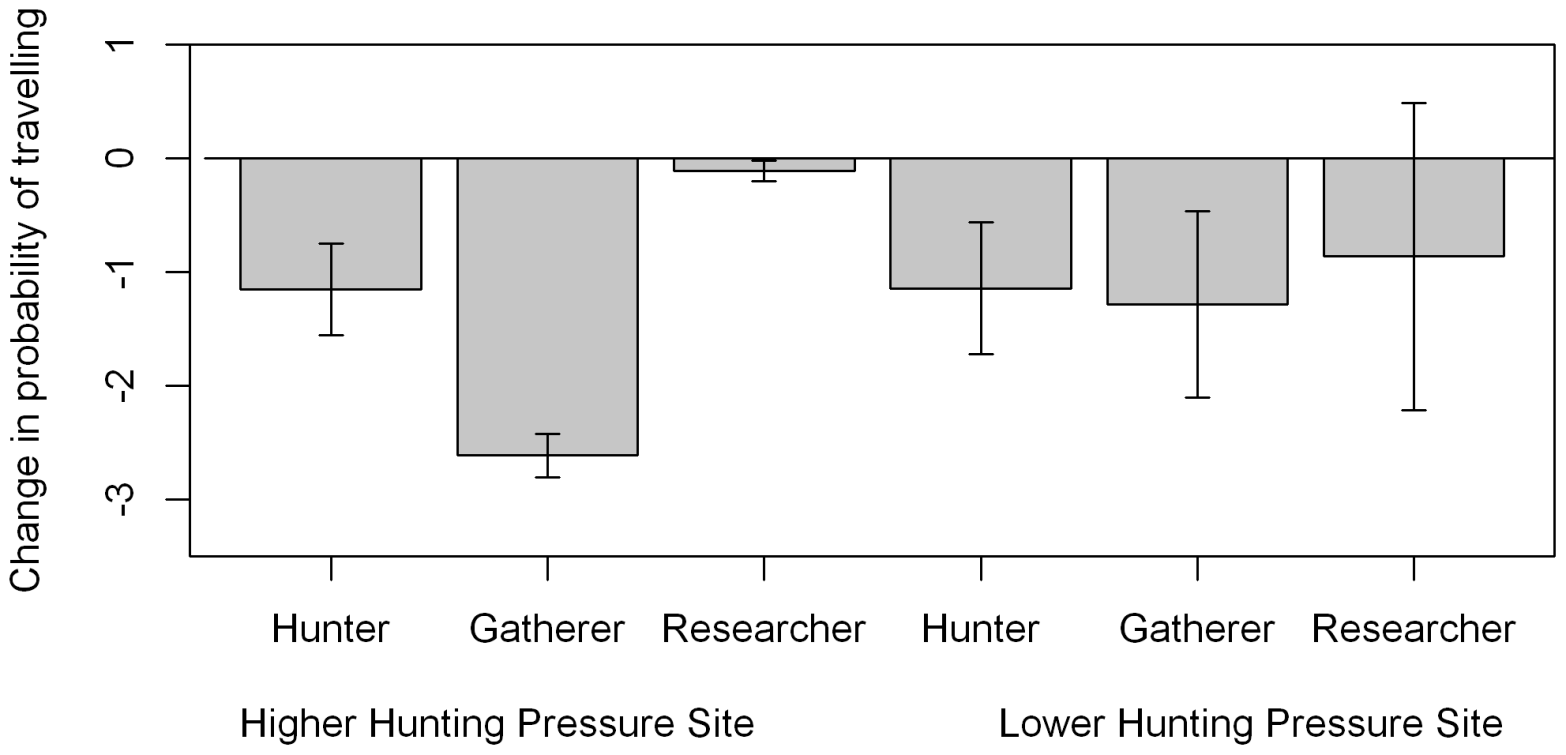
Change in travelling behaviour in the 30 minutes after experimental presentation. Change in probability of travelling (logit scale to allow standard errors to be displayed) in the 30 minutes after start of experimental presentation, compared with the previous 30 minutes. Error bars show standard errors of the estimate. P values of differences between the period before and after experimental presentation are shown: * p = 0.05–0.01, **p = 0.01–0.001, ***p<0.001. Hunter condition: Wald test, HP site: Z = 2.86, df = 1, p = 0.004; LP site: Z = 1.96, p = 0.05. Gatherer condition: Wald test, HP site: Z = 13.6, df = 1, p<0.001, LP site, p>0.05. Research condition: Wald test, both sites, p>0.05.

The best model included all interactions (Table S3), and a correlation of 0.54±0.09 between sequential observations in the same experiment. Significant decreases in travelling after experimental presentation of humans behaving as hunters was observed at both sites, and a significant decrease in travelling was also observed at the HP site in response to humans behaving as gatherers. All other experiment types showed no significant difference in travelling after experimental presentation (Figure 4).

### Change in visibility in 30 minutes after experimental presentation

**Figure 5:**
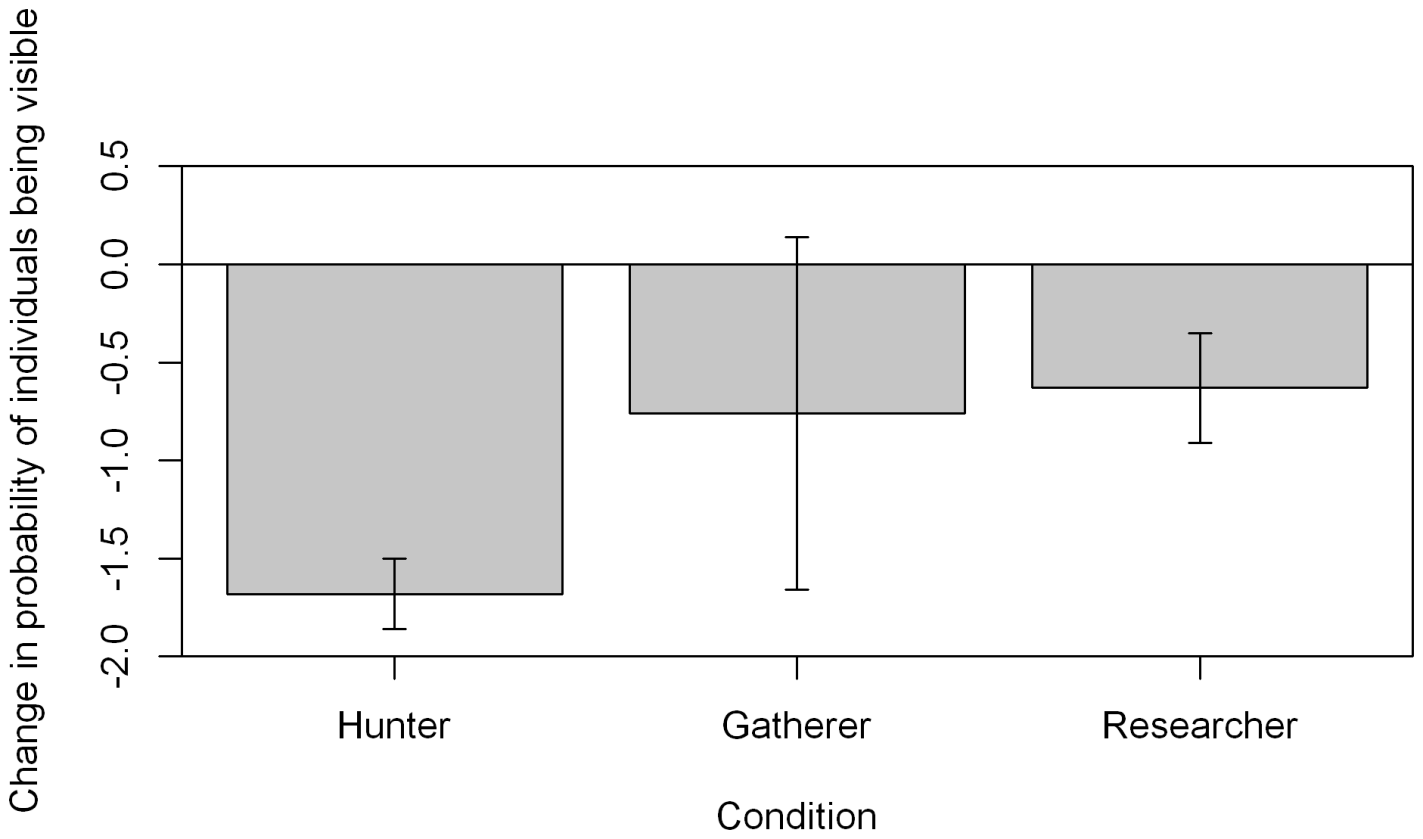
Change in visibility in the 30 minutes after experimental presentation. Change in probability any individual in the group being visible (logit scale to allow standard errors to be displayed) in the 30 minutes after start of experimental presentation, compared with the previous 30 minutes. Data from both sites is shown. Error bars show standard errors of the estimate. P values of differences between the period before and after experimental presentation are shown: * p = 0.05–0.01, **p = 0.01–0.001, ***p<0.001. Hunter condition: Wald test, Z = 9.17, df = 1, p<0.001. Researcher condition: Wald test, Z = 2.3, df = 1, p = 0.022. Gatherer condition: Wald test, p>0.05.

The best model to describe visibility during the experiment included all three main effects, an interaction between condition and time period (Table S4), and high autocorrelation of 0.58±0.06 between sequential observations in the same experiment. No interaction between site and experiment period was found, but throughout all experiments, visibility was lower at the HP site (Wald test, Z = 2.07, df = 1, p = 0.038). Visibility did not increase after experimental presentation for any condition, but only showed a significant decrease after experimental presentation of the hunter condition, and in response to the researcher condition (Figure 5).

## Disscussion

Changes in woolly monkey behaviour were observed after presentation of human models at both sites, and some degree of change was observed in response to all conditions (Table 3). However, these changes differed with both site and experimental condition, with all responses differing with condition presented. At both sites the strongest response was shown to the hunter condition, with all measures showing significant decreases in behaviour in response to experimental presentation. At the HP site, the next strongest response was to humans behaving as gatherers, with three of four measures showing decreases in observed behaviour, and the least pronounced response was to the researcher condition, where just two measures showed change; an increase in vocalisations and a decrease in visibility. In contrast, the smallest change in behaviour at the LP site was observed in response to the gatherer condition, with just an immediate increase in vocalisations, and there was a greater response to the researcher condition, with a decrease in visibility and short term decrease in vocalisations.

**Table 3:** Direction and strength of changes in behaviour after experimental presentation, and whether the observed behavioural change supports the threat sensitivity hypothesis.

Behaviour	High pressure site			Low pressure site		
	Hunter	Gatherer	Researcher	Hunter	Gatherer	Researcher
Threat level	High	Intermediate	Low	High	Intermediate	Low
Immediate vocalisations	**−2.49±1.03**	**−1.66±0.59**	−0.52±1.25	**−1.62±0.11**	**1.91±0.29**	0.34**±**0.48
Vocalisations	**−1.00±0.23**	**−0.50±0.05**	**1.70±0.26**	**−0.71±0.25**	−0.33±0.31	**−0.90±0.17**
Travel	**−1.15±0.40**	**−2.61±0.19**	−0.11±0.09	**−1.14±0.58**	−1.28±0.82	−0.86±1.35
Visibility	**−1.68±0.18**	−0.76±0.90	**−0.63±0.28**	**−1.68±0.18**	−0.76±0.90	**−0.63±0.28**
Supports hypothesis	Yes			No		

Vocal responses are displayed on a log scale, and probability of travelling and being visible are on a logit scale so standard errors can be displayed. Significant changes in behaviour are shown in bold.

Responses to the hunter condition showed a consistent decrease in all measures of behaviour at both sites. This suggests that woolly monkeys were responding to hunters cryptically, which is an appropriate response for pursuit hunters, which cannot be deterred by mobbing or other active anti-predation strategies [10]. There was also a generally cryptic response to the gatherer condition at the HP site, but unlike the response to the hunter condition, there was no significant decrease in visibility. Interestingly, although a decrease in visibility in response to the researcher condition suggested a cryptic response, an increase in vocalisations was observed in the 30 minutes after experimental presentation. This increase in vocalisations may be an increase in contact calls as individuals confirm the location of other group members [41]. A short-term increase in vocalisations was also observed at the LP site, but in response to the gatherer condition. Responses to the researcher condition at the LP site again suggested a cryptic response, with vocalisations and visibility decreasing.

Results at the HP site are in agreement with the predicted threat sensitive predator response, as a stronger change in behaviour was observed in response to more threatening humans. Although monkeys at the LP also showed stronger responses to the hunter condition, they did not show the predicted weaker response to humans behaving as researchers than humans behaving as gatherers. This may be because monkeys at the LP site are naive, and have insufficient experience with the three types of human presented to respond as predicted. Nevertheless, reactions to hunters were consistent with reactions at the HP site and reactions to each condition were distinct, so woolly monkeys at the LP do not appear to assess all humans in the same way. Alternately, woolly monkeys at the LP site may lack experience specifically with gatherers, and so do not respond appropriately to their presence. Gatherers use areas closer to the community and make shorter trips [42], so it is plausible that woolly monkeys at the LP site (more than 10 km from the nearest settlement, compared with around 2 km at the HP site) are less exposed to gatherers. Likewise, the HP site generally has fewer researchers, and most of these work in a single 50ha plot. This lack of knowledge may explain the paradoxical increase in vocal response at each site, as group members may vocalise in response to the novel condition.

An alternate explanation for this unexpected result at the LP site may be that relative threat for each human condition is not consistent for the two sites. At the HP site, it is not unreasonable to make the assumptions of this study in terms of the relative lethal threat posed by each condition – hunters are immediately and lethally threatening, gatherers are potentially lethally threatening, and researchers pose no mortal threat. However, at the LP site, darting of various primate species, including numerous woolly monkeys, has occurred to attach radio collars, and several dozen woolly monkeys in the area have had small amounts of tissue extracted with non-lethal biopsy darts [43], [44]. As a result of this, researchers could be considered greater threats than gatherers at the LP site, because being hit by a biopsy dart may evoke a strong reaction even though it is non-lethal. By contrast, only two woolly monkey females, and members of no other species, have experienced biopsy darts in the HP study area, and this occurred in 1998 [44]. Researchers may therefore be perceived as more threatening than gatherers for woolly monkeys at the LP site due to the higher levels of darting which have occurred. Quantitative data on the response of woolly monkeys shot with a poisoned arrow or biopsy darts are unavailable, but if these responses are similar, non-targeted individuals may associate the auditory and visual cues from the shot monkey with the presence of both hunters and researchers. Previous research on primates and other mammals has shown that social learning about predators is possible [19], and if woolly monkeys can learn socially, they need not personally experience a predation threat. Although this is speculative, if these, or other similarities exist, woolly monkeys at the LP site may be showing a response consistent with perceived threat levels. Biopsy darting no longer occurs at the LP site due to advances in DNA extraction techniques, but as woolly monkeys can live up to 30 years [45], those who have experienced this procedure may still present in the population. Although experiments were not conducted on collared individuals, the darting took place on various groups at the LP site, and individuals who experienced darting may have migrated to groups with which these experiments were conducted.

These results could explain previously noted variability in primate reactions to humans. Although previous studies have suggested this variability is due to spatial variation in hunting pressure [11], [14], reactions to humans in these studies were not consistent even within a single area. In this study, woolly monkeys showed different responses both with spatial variation in hunting pressure and with the type of stimulus presented. Woolly monkeys generally responded cryptically to the stimuli, but an increase in vocalisations was observed to the possible least threatening condition at each site. Previous studies [10], [12], [13] do not give details about the behaviour of their human models, but variations in behaviour of these human models in their studies could have resulted in the differing responses observed. The unexpected responses of fleeing and vocalising [14] could be because monkey groups were correctly assessing those conducting the research as researchers.

From the results of this study, it is unclear which cues woolly monkeys use to distinguish different classes of humans. Previous research on semi-wild Colombian black-tailed deer responses to hunters showed deer fled further when approached more quickly and directly [46], which could be a general response to large predators and not human-specific. In this study, the behaviours associated with each condition were designed to simulate the differences between hunters, gatherers and researchers, rather than determine which cues are used by monkey groups. K. Zuberbühler (personal communication) suggested that groups respond differently based on the gaze direction of humans, but this hypothesis is not supported by the observed results in this study. It is possible that the large, 2.4m blowpipe is a reliable cue for hunters, but it is unclear which cues distinguish researchers and gatherers as the equipment they carry is smaller and far more variable in natural encounters (for example, researchers working on small terrestrial animals may be unlikely to carry binoculars).

Primate reactions to non-human predators are well studied, but most studies have contrasted reactions to different predator species (e.g. blue monkey *Cercopithecus mitis* reactions to simulated leopards and eagles [47]), rather than differing responses to a single species. These results suggest that members of hunted primate populations can use the behaviour of humans to distinguish between dangerous and non-dangerous individuals, and respond less strongly to lower threats. This ability reduces the potential negative impacts of antipredator behaviours on prey species. This ability is not only important when a species is frequent but attacks are not, but also when food or other resources are limiting for a population. These experiments only cover a short period after the presentation of a potential predator, but the impacts of anti-predator behaviour can be significant. Primates in areas where hunting occurs can freeze for up to five hours after encountering humans (F. Maisels, personal communication). It is worth noting that all humans had some effect on woolly monkeys, including researchers whose intentions are benign. The presence of any human could affect the behaviour of hunted primate populations, which may have implications for both academic research and the conservation of these species. In particular, the possible consequences of human presence and research on species, especially those threatened by hunting, should be carefully considered during the initial stages of any project.

## Acknowledgments

This research was approved by the Imperial College Ethics Committee, and the authors are grateful for the approval and research permit from the Ministerio del Ambiente, Provincial de Orellana, Ecuador. Particular thanks to the staff at Yasuní Research Station, Tiputini Biodiversity Station and Proyecto Primates for their support, Antony Di Fiore and two reviewers for their comments and suggestions, and to the field assistants Cristina Porras and Andrea Salcedo.

## Supporting Information

Table S1 **QICu and ΔQICu of generalised estimating equations with number of calls per minute in the periods immediately before, during and immediately after experimental presentation as a dependant variable (n = 315 in 21 experiments).**
(DOCX)

Table S2 **QICu and ΔQICu of generalised estimating equations with number of calls per 5 minute block throughout the one hour experiment as a dependant variable (n = 252 in 21 experiments).**
(DOCX)

Table S3 **QICu and ΔQICu of generalised estimating equations presence/absence of travelling per 5 minute block throughout the one hour experiment as a dependant variable (n = 252 in 21 experiments).**
(DOCX)

Table S4 **QICu and ΔQICu of generalised estimating equations presence/absence of visible individuals per 5 minute block throughout the one hour experiment as a dependant variable (n = 252 in 21 experiments).**
(DOCX)
