# Correction: Hunted Woolly Monkeys (*Lagothrix poeppigii*) Show Threat-Sensitive Responses to Human Presence

**DOI:** 10.1371/annotation/2adfbe03-9c3a-4df1-ae04-1ea05abeb4e0

**Published:** 2013-09-12

**Authors:** Sarah Papworth, E. J. Milner-Gulland, Katie Slocombe

The authors wish to add the following sentence to the Copyright Statement:

"Please contact the corresponding author for information on the underlying research materials." 

