# Supplementary material for: Hunted Woolly Monkeys (Lagothrix poeppigii) Show Threat-Sensitive Responses to Human Presence
Source: PLoS One. 2013 Apr 16;8(4):e62000. doi: 10.1371/journal.pone.0062000 (PMC3629061; doi:10.1371/journal.pone.0062000)
Supplement: Table S1 — QICu and ΔQICu of generalised estimating equations with number of calls per minute in the periods immediately before, during and immediately after experimental presentation as a dependant variable (n = 315 in 21 experiments). (DOCX) [file pone.0062000.s001.docx]

Table S1: QICu and ΔQICu of generalised estimating equations with number of calls per minute in the periods immediately before, during and immediately after experimental presentation as a dependant variable (n=315 in 21 experiments).

| **Model** | **QICu** | **ΔQICu** |
| --- | --- | --- |
| S^[[1]](#footnote-1)^ + C^[[2]](#footnote-2)^ + P^[[3]](#footnote-3)^ + SxP + CxP + SxC + SxCxP | 200.00 | 0.00 |
| S + C + P + SxP + CxP | 262.20 | 62.20 |
| S + C + P + CxP | 281.30 | 81.30 |
| C + P + CxP | 557.90 | 357.90 |
| S + P + SxP | 575.20 | 375.20 |
| S + P | 685.30 | 485.30 |
| S | 729.38 | 529.38 |
| P | 891.70 | 691.70 |
| Null | 995.12 | 795.12 |

1. Site [↑](#footnote-ref-1)
2. Condition [↑](#footnote-ref-2)
3. Period [↑](#footnote-ref-3)
