# Supplementary material for: Hunted Woolly Monkeys (Lagothrix poeppigii) Show Threat-Sensitive Responses to Human Presence
Source: PLoS One. 2013 Apr 16;8(4):e62000. doi: 10.1371/journal.pone.0062000 (PMC3629061; doi:10.1371/journal.pone.0062000)
Supplement: Table S2 — QICu and ΔQICu of generalised estimating equations with number of calls per 5 minute block throughout the one hour experiment as a dependant variable (n = 252 in 21 experiments). (DOCX) [file pone.0062000.s002.docx]

Table S2: QICu and ΔQICu of generalised estimating equations with number of calls per 5 minute block throughout the one hour experiment as a dependant variable (n=252 in 21 experiments).

| **Model** | **QICu** | **ΔQICu** |
| --- | --- | --- |
| S^[[1]](#footnote-1)^ + C ^[[2]](#footnote-2)^+ P^[[3]](#footnote-3)^ + SxP + CxP + SxC + SxCxP | -7446.50 | 0.00 |
| S + C + P + SxP + CxP | -7211.10 | 235.40 |
| S + C + P + CxP | -7135.60 | 310.90 |
| S + P + SxP | -6896.27 | 550.23 |
| S + P | -6834.36 | 612.14 |
| C + P + CxP | -6817.00 | 629.50 |
| S | -6747.34 | 699.16 |
| P | -6505.25 | 940.25 |
| Null | -6427.19 | 1019.31 |

1. Site [↑](#footnote-ref-1)
2. Condition [↑](#footnote-ref-2)
3. Period [↑](#footnote-ref-3)
