# Supplementary material for: Hunted Woolly Monkeys (Lagothrix poeppigii) Show Threat-Sensitive Responses to Human Presence
Source: PLoS One. 2013 Apr 16;8(4):e62000. doi: 10.1371/journal.pone.0062000 (PMC3629061; doi:10.1371/journal.pone.0062000)
Supplement: Table S3 — QICu and ΔQICu of generalised estimating equations presence/absence of travelling per 5 minute block throughout the one hour experiment as a dependant variable (n = 252 in 21 experiments). (DOCX) [file pone.0062000.s003.docx]

Table S3: QICu and ΔQICu of generalised estimating equations presence / absence of travelling per 5 minute block throughout the one hour experiment as a dependant variable (n=252 in 21 experiments).

| **Model** | **QICu** | **ΔQICu** |
| --- | --- | --- |
| S^[[1]](#footnote-1)^ + C^[[2]](#footnote-2)^ + P^[[3]](#footnote-3)^ + SxP + CxP + SxC + SxCxP | 785.7 | 0.00 |
| S + C + P + SxP + CxP + SxC | 797.7 | 12.00 |
| S + C + P + SxP + CxP | 797.8 | 12.10 |
| C + P + CxP + SxC | 815.2 | 29.50 |
| S + P + SxP | 817 | 31.30 |
| S + P | 817.5 | 31.80 |
| P | 833.58 | 47.90 |
| S | 835.21 | 49.50 |
| Null | 850.23 | 64.50 |

1. Site [↑](#footnote-ref-1)
2. Condition [↑](#footnote-ref-2)
3. Period [↑](#footnote-ref-3)
