# Supplementary material for: Hunted Woolly Monkeys (Lagothrix poeppigii) Show Threat-Sensitive Responses to Human Presence
Source: PLoS One. 2013 Apr 16;8(4):e62000. doi: 10.1371/journal.pone.0062000 (PMC3629061; doi:10.1371/journal.pone.0062000)
Supplement: Table S4 — QICu and ΔQICu of generalised estimating equations presence/absence of visible individuals per 5 minute block throughout the one hour experiment as a dependant variable (n = 252 in 21 experiments). (DOCX) [file pone.0062000.s004.docx]

Table S4: QICu and ΔQICu of generalised estimating equations presence / absence of visible individuals per 5 minute block throughout the one hour experiment as a dependant variable (n=252 in 21 experiments).

| **Model** | **QICu** | **ΔQICu** |
| --- | --- | --- |
| S^[[1]](#footnote-1)^ + C^[[2]](#footnote-2)^ + P^[[3]](#footnote-3)^ + CxP | 756.9 | 00.00 |
| S + C + P + SxP + CxP | 757.0 | 00.10 |
| S + P + SxP | 769.0 | 12.10 |
| S + P | 769.54 | 12.64 |
| C + P + CxP | 770.0 | 13.10 |
| S | 779.28 | 22.38 |
| P | 782.5 | 23.60 |
| Null | 791.73 | 32.83 |
| S + C + P + SxP + CxP + SxC + SxCxP | Singular | NA |

1. Site [↑](#footnote-ref-1)
2. Condition [↑](#footnote-ref-2)
3. Period [↑](#footnote-ref-3)
